# Differences in chronic kidney disease management based on identification and diagnosis in a population-based observational study

**DOI:** 10.1007/s40620-025-02414-2

**Published:** 2025-09-26

**Authors:** Michael Olszewski, Karl Bjurström, Markus Lingman, Dan Henrohn, Poyan Shojaiyan, Magnus Garell, Björn Agvall

**Affiliations:** 1 Department of Research and Development, Region Halland, Halmstad, Sweden; 2 Primary Healthcare, Region Halland, Halmstad, Sweden; 3 Halland Hospital, Region Halland, Halmstad, Sweden; 4 Center for Applied Intelligent Systems Research, School of Information Technology, Halmstad University, Halmstad, Sweden; 5 Department of Molecular and Clinical Medicine, Institute of Medicine, Sahlgrenska Academy, University of Gothenburg, Göteborg, Sweden; 6 AstraZeneca AB, Stockholm, Sweden; 7 Department of Medical Sciences, Uppsala University, Uppsala, Sweden; 8 Center for Primary Health Care Research, Department of Clinical Sciences, Malmö, Lund University, Malmö, Sweden

**Keywords:** Chronic kidney disease, Epidemiology, Disease management, International classification of diseases

## Abstract

**Background:**

Chronic kidney disease (CKD) affects 6–10% of adults and often remains undiagnosed until advanced stages, leading to inadequate management. This study compared diagnosed, proxy-diagnosed, and undiagnosed CKD patients regarding prevalence, clinical assessment, nephroprotective treatment, healthcare utilization, and mortality.

**Methods:**

This retrospective observational study analyzed Region Halland's healthcare data for adults meeting KDIGO CKD-confirmed criteria for the year 2019. Patients were categorized as diagnosed CKD (ICD-coded), proxy-diagnosed CKD (CKD-related diagnoses), or undiagnosed CKD (meeting CKD criteria without an ICD CKD diagnosis).

**Results:**

Of 20,488 CKD patients, 21% had diagnosed CKD, 18% proxy-diagnosed CKD, and 61% undiagnosed CKD. Mean ages were 76.4, 62.4, and 81.8 years, respectively (*p* < 0.001). Blood pressure follow-up was carried out in diagnosed CKD (88%) versus 67% and 80% in the proxy-diagnosed and undiagnosed groups. eGFR was tested in 66% overall (73% diagnosed, 53% proxy-diagnosed, 66% undiagnosed), while urine albumin-to-creatinine ratio (UACR) testing was performed in 27% overall (50%, 20%, and 21%, respectively). Renin-angiotensin system inhibitors were prescribed to 45% overall (51%, 28%, and 47%, respectively). The adjusted hospitalization risk was 2.71 (CI: 2.59–2.84) in diagnosed CKD and 1.38 (CI: 1.31–1.46) in proxy-diagnosed CKD. Adjusted all-cause mortality hazard ratios were 2.22 (CI: 1.95–2.52) and 1.31 (CI: 1.08–1.60), respectively. Stratified sensitivity analyses by CKD stage confirmed these associations, though the strength varied.

**Conclusions:**

Patients with complex comorbidities, more advanced CKD, and frequent hospitalizations are more likely to be diagnosed with CKD and receive better follow-up care. Proxy-diagnosed CKD was common and associated with suboptimal management. These findings emphasize the need for consistent and accurate CKD identification to improve outcomes and optimize care.

**Graphical abstract:**

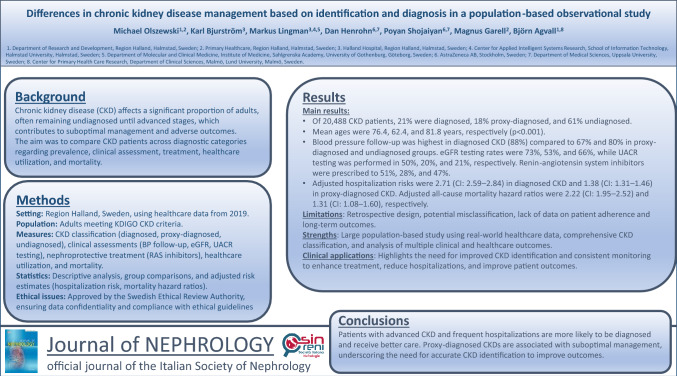

**Supplementary Information:**

The online version contains supplementary material available at 10.1007/s40620-025-02414-2.

## Introduction

Chronic kidney disease (CKD) affects an estimated 850 million people worldwide and is characterized by a persistent decrease in kidney function or structural abnormalities lasting more than three months [1, 2]. Among adults, the prevalence of CKD ranges from 6 to 10% [1–3]. The primary drivers of CKD include diabetes mellitus and hypertension, while other significant contributors are atherosclerotic cardiovascular disease (ASCVD), glomerulonephritis, and autosomal dominant polycystic kidney disease [4, 5]. In 2017, CKD-related cardiovascular diseases caused 1.4 million deaths, with CKD solely responsible for 1.2 million fatalities. By 2040, CKD-related deaths are projected to reach between 2.2 and 3 million, posing a significant economic burden on healthcare systems [4, 6].

Interventions with renin-angiotensin-aldosterone system inhibitors (RASi) and sodium-glucose cotransporter-2 inhibitors (SGLT2i) may help slow the decline in kidney function, and aim to prevent the need for future dialysis [7–9]. Statins are also important in patients over 50 years, as ASCVD is a common cause of CKD [10]. Current guidelines recommend that individuals aged 50 or older with an estimated glomerular filtration rate (eGFR) below 60 ml/min/1.73 m^2^ on two separate occasions, at least three months apart, should receive lipid-lowering medication to prevent cardiovascular disease, regardless of their cholesterol levels [10, 11]. Statins consistently reduce cardiovascular risk, regardless of initial LDL cholesterol levels.

The detection of CKD in a patient often occurs incidentally, as early CKD symptoms are sparse and nonspecific. Typically, symptoms related to CKD manifest only after a significant decline in kidney function, usually below 20 ml/min/1.73m^2^. Both reduced eGFR and albuminuria are distinct factors predicting CKD progression and moreover, the presence of albuminuria concurrently with reduced eGFR tends to hasten the decline in kidney function [12]. It is recommended that patients with diabetes mellitus, hypertension, cardiovascular diseases, and those who have undergone nephrectomy should check kidney function at least once a year according to the international KDIGO guidelines for CKD [11, 13]. In the case of newly discovered renal impairment, a more frequent control regimen with subsequent tapering is initially recommended to monitor the rate of kidney function loss. A decline in eGFR, characterized by a decrease of >15% over 3 months or >25% within a year, after excluding pre- or post-renal causes, indicates a rapid progression in CKD and should prompt a nephrological assessment and more intense follow-up [11]. Referral to a nephrologist is recommended when the urine albumin-to-creatinine ratio (UACR) exceeds 30 mg/mmol, as this indicates significant kidney damage. This threshold is widely adopted in many countries to ensure timely specialist intervention [11]. Despite this, it was possible to demonstrate in two Swedish studies that measurement of urine UACR was only performed on 24-27% of patients with CKD, and in 38% of patients with diabetes [3, 10]. A comparable trend has been observed with annual follow-up monitoring of UACR in hypertension, with a rate of 29% [13].

Previous research from Region Halland has revealed that only 18% of cases receive an International Classification of Diseases (ICD) code for CKD [12]. This finding aligns with previous research indicating a similarly low rate of ICD diagnosis for the disease [3, 15]. However, in our cohort there were patients having a diagnosed CKD, those having an ICD diagnosis that was indirectly related to CKD (proxy-diagnosed CKD), and those without an ICD diagnosis despite having CKD according to KDIGO criteria. A proxy-diagnosed CKD refers to the identification of CKD through diagnoses that are not explicitly classified as CKD but are strongly linked to the condition, such as diabetes with kidney complications or a history of glomerulonephritis. Individuals with declining kidney function are more likely to receive an ICD diagnosis for CKD, particularly at more advanced stages of the disease [12]. Research indicates that having a documented CKD diagnosis is associated with significant improvements in disease management and monitoring practices, a lower risk of major adverse cardiovascular events, a lower risk of kidney failure (transplant or dialysis), and a slower decline in eGFR [16, 17]. Early detection of CKD is essential, as it enables timely treatment interventions that can help slow disease progression. In this context, it was also important for patients to be aware of their condition. However, patient awareness of CKD was reportedly low, which was largely influenced by the terminology healthcare providers used and how the diagnosis was recorded [18].

The aim of the study was to explore, in a population of patients with CKD, the differences between those with diagnosed CKD, proxy-diagnosed CKD and those with undiagnosed CKD, to describe the differences between these groups regarding prevalence, follow-up, nephroprotective treatment, and to identify whether there are any differences in healthcare utilization and mortality.

## Methods

### Setting

This retrospective observational study was based on a CKD population in Region Halland and included registry data from Region Halland's data system, the Regional Healthcare Information Platform (RHIP) [19]. Region Halland is located in southwestern Sweden and has a population of 340,000 inhabitants. During the time period considered in this study, it had a healthcare infrastructure that included three hospitals, 40 inpatient wards, two emergency departments, 30 specialist outpatient clinics, and 46 primary care clinics. Of these primary care units, 23 were privately administered, and 23 were under public administration.

### Data source

Region Halland utilizes the RHIP database, which offers pseudo-anonymized data encompassing clinical, operational, and financial information for all individuals treated since 2011 in both public and private healthcare facilities within the region [19]. This study relied on RHIP as its primary data source, a method previously used in research on heart and kidney failure populations in Region Halland. RHIP consolidated data from various healthcare services, including primary care, emergency services, hospital admissions, outpatient, and inpatient care. It offered a comprehensive view of the patient population in Region Halland, linking clinical, operational, and cost data at the level of individual encounters, and included details on healthcare system resources and capacity, such as the availability of nurses, doctors, and hospital beds. RHIP records included data on deceased patients, noting the date of death, which enabled analysis of overall mortality within the cohort. Prescribed medication information was obtained from the National Pharmacy Register.

### Study population

The research included individuals aged 18 and older with impaired kidney function, as defined by KDIGO guidelines [11]. The primary criterion for inclusion was a sustained reduction in eGFR below 60 mL/min/1.73 m^2^ on two separate occasions, with at least a 90-day gap between tests, as detailed in Supplementary Table 1 [20–22]. Alternatively, eligibility also included individuals diagnosed with kidney disease via an ICD code indicative of CKD or those with elevated UACR, when such data were available (Supplement Table 1). All study subjects were required to be residents of Region Halland for the duration of the study and to have received any registered healthcare service within the region. The cohort selection process is illustrated in Supplementary Figure 1.

**Table 1:** Characteristics of individuals with diagnosed CKD, proxy-diagnosed CKD, and undiagnosed CKD

	Diagnosed	Proxy-diagnosed	Undiagnosed	Total	*p*-value
	CKD	CKD	CKD	cohort	
Total cohort, *n* (%)	4323	3659	12,506	20,488	
*CKD stages, n (%)*					
Not applicable	48 (1)	573 (16)	2 (<1)	623 (3)	<0.001^1^
CKD stage 1	73 (2)	619 (17)	20 (<1)	712 (4)	
CKD stage 2	492 (11)	1566 (43)	1597 (13)	3655 (18)	
CKD stage 3a	1041 (24)	647 (18)	7815 (62)	9503 (46)	
CKD stage 3b	1398 (32)	215 (6)	2722 (22)	4335 (21)	
CKD stage 4	1036 (24)	37 (1)	345 (3)	1418 (7)	
CKD stage 5	235 (5)	2 (<1)	5 (<1)	242 (1)	
*Gender, n (%)*					
Female	1761 (41)	2170 (59)	7121 (57)	11052 (54)	<0.001^1^
Male	2562 (59)	1489 (41)	5385 (43)	9436 (46)	
Age, mean (SD)	76.4 (13.6)	62.4 (19.4)	81.8 (8.7)	77.2 (14.3)	<0.001^2^
*Age groups, n (%)*					
18‒54 years	335 (8)	1158 (32)	98 (1)	1591 (8)	<0.001^1^
55‒64 years	347 (8)	550 (15)	307 (2)	1204 (6)	
65‒74 years	847 (20)	819 (22)	1853 (15)	3519 (17)	
75‒84 years	1508 (35)	732 (20)	5213 (42)	7453 (36)	
85‒94 years	1124 (26)	358 (10)	4368 (35)	5850 (29)	
>95 years	162 (4)	42 (1)	667 (5)	871 (4)	
*Comorbidities, n (%)*					
Diabetes mellitus	1399 (32)	622 (17)	2244 (18)	4265 (21)	<0.001^1^
Heart failure	1335 (31)	233 (6)	1803 (14)	3371 (16)	<0.001^1^
Hypertension	3569 (83)	1573 (43)	9383 (75)	14525 (71)	<0.001^1^
ASCVD	1623 (38)	598 (16)	3266 (26)	5487 (27)	<0.001^1^
No comorbidity	450 (10)	1794 (49)	2223 (18)	4467 (22)	<0.001^1^
1 comorbidity	1294 (30)	988 (27)	5495 (44)	7777 (38)	
2 comorbidities	1409 (33)	626 (17)	3373 (27)	5408 (26)	
≥3 comorbidities	1170 (27)	251 (7)	1415 (12)	2836 (14)	
*Pharmacotherapy, n (%)*					
Statins	1886 (44)	749 (21)	4404 (35)	7039 (34)	<0.001^1^
SGLT-2i	34 (1)	44 (1)	89 (1)	167 (1)	0.01^1^
ACEis	1008 (23)	488 (13)	2837 (23)	4333 (21)	<0.001^1^
ARBs	1197 (28)	541 (15)	3101 (25)	4839 (24)	<0.001^1^
RASis	2183 (50)	1019 (28)	5891 (47)	9093 (44)	<0.001^1^
Beta-blockers	2291 (53)	797 (22)	5482 (44)	8570 (42)	<0.001^1^
Calcium antagonists	1457 (34)	505 (14)	3307 (26)	5269 (26)	<0.001^1^
*UACR*					
A1	1501 (35)	658 (18)	2319 (18)	4478 (22)	0.001
A2	592 (14)	82 (2)	240 (2)	914 (4)	
A3	77 (<1)	7 (<1)	6 (<1)	90 (<1)	

*CKD* chronic kidney disease, *n* number, *SD* standard deviation, *ASCVD* atherosclerotic cardiovascular disease, *SGLT-2i* sodium-glucose cotransporter-2 inhibitor, *ACEi* angiotensin-converting enzyme inhibitor, *ARB* angiotensin receptor blocker, *RASi* renin-angiotensin system inhibitors, *UACR* urine albumin-to-creatinine ratio

^1^Pearson Chi-Square

^2^One-way ANOVA

The patients were categorized into three groups. The first group, defined as “Diagnosed CKD”, included those with a verified ICD diagnosis of CKD. The second group, called “Undiagnosed CKD,” consisted of individuals who did not have any CKD or CKD-related ICD diagnosis but met the inclusion criteria for CKD. Specifically, undiagnosed CKD was defined as having a reduced eGFR on two separate occasions at least 90 days apart and/or elevated UACR, without any registered ICD-10 diagnosis indicating CKD. The third group, called “proxy-diagnosed CKD”, comprised patients with a CKD-related ICD diagnosis used as a proxy to indicate CKD. The diagnostic criteria for “diagnosed CKD” and “proxy-diagnosed CKD” are detailed in Supplementary Table 2. The proxy-diagnosed CKD group included individuals without a formal CKD diagnosis but with ICD-10 codes for conditions strongly associated with CKD, such as diabetic nephropathy, hypertensive nephrosclerosis, or glomerulonephritis. These codes were selected based on established associations with CKD in previous literature, enabling identification of likely but undocumented cases. The study covers the period from January 2019 to December 2019.

### Study process

All individuals diagnosed with impaired kidney function according to KDIGO guidelines from 2013 to 2019 were recorded. The study included those with renal impairment who were alive at any point in 2019. For these individuals, details such as age, gender, and comorbidities were documented (Supplementary Table 2). Laboratory data from 2019, including eGFR, glycated hemoglobin (HbA1c), and cholesterol levels (total cholesterol and LDL-cholesterol), were collected. These variables were selected due to their clinical relevance to CKD diagnosis, staging, and management, as well as their consistent availability across the study population. Patients’ blood pressure readings for 2019 were also gathered, with the average value used in cases where multiple readings were available. All available albuminuria data were collected to determine albuminuria levels. The study tracked healthcare utilization by recording the number of hospital admissions and the total days of hospitalization for the year 2019. The number of visits to see nurses and physicians in outpatient settings, including hospital outpatient departments, primary care facilities, and emergency departments was registered. Pharmacological treatments for blood pressure, diabetes, and cholesterol management were identified, with the associated ATC codes provided in Supplementary Table 3. Deaths occurring during the study period were documented to calculate the one-year mortality rates across the cohorts.

### Statistics

Data were summarized using descriptive statistics. Mean values and standard deviations (SD) were used to represent continuous data, which were analyzed by Student’s t-test. The one-way ANOVA test was employed to compare means between multiple groups. Frequencies and percentages of categorical data were analyzed by the Chi-square test. Kidney function was classified into the 5 KDIGO stages, and patients without eGFR data for 2019 were labeled as “not applicable” (NA). Age groups were divided into six categories: 18-54, 55-64, 65-74, 75-84, 85-94, and >95 years.

Hospitalization data were analyzed with negative binomial regression, using the number of hospital days as the outcome variable, and adjusting for ICD diagnosis status, age, sex, CKD stages, and comorbidities. The CKD stages were compiled into three groups: CKD stages 1-2 in the first group, CKD stage 3 in the second, and CKD stages 4-5 in the third. Cox regression analysis for mortality was also performed, adjusting for ICD diagnosis status, age, sex, CKD stage (grouped as previously mentioned), and comorbidities. To assess the robustness of these associations, additional sensitivity analyses were conducted, stratified by CKD stage group, for both hospitalization and mortality outcomes. These models were used to evaluate whether the associations between diagnosis status and outcomes remained consistent across different levels of kidney function and were summarized using forest plots. All statistical analyses used a two-sided test approach, with a p-value of less than 0.05 indicating significant differences. IBM SPSS Statistics Version 29.0, Armonk, New York, USA was used for the analysis.

## Results

The total study cohort consisted of 20,488 individuals who met the criteria for CKD according to the KDIGO confirmed criteria. Among these individuals, 4323 (21%) had a diagnosed CKD documented according to ICD codes, 3659 (18%) had a proxy-diagnosed CKD, and 12,506 (61%) had an undiagnosed CKD but had CKD based on laboratory criteria.

There were 2794 (14%) individuals in the diagnosed CKD group aged ≥75 years. In the group with proxy-diagnosed CKD, 1132 (6%) belonged to this age range, while the group with undiagnosed CKD comprised 10,248 (50%) patients in this age category. In the diagnosed CKD group, 60% had two or more comorbidities, compared to 24% in the proxy-diagnosed CKD group and 39% in the undiagnosed CKD group. The basic characteristics including CKD stages, comorbidities and pharmacotherapy are displayed in Table 1.

Statins were prescribed to 34% of patients, including 44% with diagnosed CKD, 21% with proxy-diagnosed CKD, and 35% in the undiagnosed CKD group. RASi pharmacotherapy (Angiotensin Receptor Blockers [ARBs] or Angiotensin Converting Enzyme inhibitors [ACEi]) was used by 44% of the cohort, with 50% in the diagnosed CKD group, 28% in the proxy-diagnosed group, and 47% in the undiagnosed group. A total of 79 patients received both ARBs and ACEi during the study. Screening with UACR was low overall (27%, Table 2). Among those tested for UACR, the distribution into A1, A2 and A3 was; 69.2%, 27.3% and 3.5% in the diagnosed CKD group, 88.1%, 11% and 0.9% in the proxy-diagnosed CKD group, and 90.4%, 9.4% and 0.2% in the undiagnosed CKD group. Calculation of the distribution of UACR levels (A1, A2 or A3) based on total cohort data are presented in Table 1.

**Table 2:** Total healthcare utilization in 2019

	Diagnosed CKD	Proxy-diagnosed CKD	Undiagnosed CKD	Total cohort	*p*-value
*Total cohort, n*	4323	3659	12,506	20,488	
*Healthcare utilization*					
*Hospital inpatient care*					
Admissions, *n* (%)	1852 (43)	910 (29)	2974 (24)	5736 (28)	<0.001^1^
Admissions, mean (SD)	0.9 (1.5)	0.4 (1.0)	0.4 (0.8)	0.5 (1.1)	<0.001^2^
Inpatient care days, mean (SD)	5.6 (12.4)	2.1 (7.3)	2.0 (6.3)	2.8 (8.3)	<0.001^2^
*Hospital outpatient care*					
Emergency department, mean (SD)	0.5 (0.5)	0.3 (0.5)	0.3 (0.5)	0.3 (0.5)	<0.001^2^
Physician, mean (SD)	0.7 (0.5)	0.6 (0.5)	0.5 (0.5)	0.6 (0.5)	<0.001^2^
Nurse, mean (SD)	0.6 (0.5)	0.3 (0.5)	0.3 (0.5)	0.4 (0.5)	<0.001^2^
*Primary care*					
Physician, mean (SD)	0.9 (0.4)	0.8 (0.4)	0.9 (0.3)	0.9 (0.3)	<0.001^2^
Nurse, mean (SD)	0.9 (0.3)	0.8 (0.4)	0.9 (0.3)	0.9 (0.3)	<0.001^2^
*Number of admissions*					
0, *n* (%)	2471 (57)	2749 (75)	9532 (76)	14752 (72)	<0.001^1^
1, *n* (%)	935 (22)	583 (16)	2047 (16)	3565 (17)	
2, *n* (%)	447 (10)	185 (5)	581 (5)	1213 (6)	
3, *n* (%)	211 (5)	75 (2)	191 (2)	477 (2)	
4, *n* (%)	127 (3)	23 (1)	85 (1)	235 (1)	
5, *n* (%)	50 (1)	19 (1)	30 (<1)	99 (1)	
>5, *n* (%)	82 (1)	25 (1)	40 (<1)	147 (1)	
*Follow-up testing*					
Blood pressure, *n* (%)	3809 (88)	2462 (67)	9955 (80)	16226 (79)	<0.001^1^
Glucose test, *n* (%)	3581 (83)	2513 (69)	9536 (76)	15630 (76)	<0.001^1^
eGFR, *n* (%)	3175 (73)	1938 (53)	8253 (66)	13366 (65)	<0.001^1^
UACR, *n* (%)	2170 (50)	747 (20)	2565 (21)	5482 (27)	<0.001^1^
Urine (Dipstick), *n* (%)	2266 (52)	1656 (45)	4705 (38)	8627 (42)	<0.001^1^
No proteinuria test, *n* (%)	1392 (32)	1739 (48)	6827 (55)	9958 (49)	<0.001^1^

*CKD* chronic kidney disease, *eGFR* estimated glomerular filtration rate, *UACR* urine albumin-to-creatinine ratio, *SD* standard deviation, *n* number

^1^Pearson Chi-Square

^2^One-way ANOVA

The data showed an average of 0.9 (1.5) hospital admissions and 5.6 (12.4) days per hospital stay for patients with diagnosed CKD. This contrasts with the proxy-diagnosed CKD diagnosis group, which averaged 0.4 (1.0) hospital admissions and 2.1 (7.3) days per hospital stay. In 2019, among those with no hospital admissions, there were 9532 (76%) individuals in the undiagnosed CKD group, 2471 (57%) in the diagnosed CKD group, and 2749 (75%) in the proxy-diagnosed CKD group. Details on healthcare utilization are provided in Table 2.

In 2019, annual monitoring of key health parameters varied across the different CKD groups. Within the diagnosed CKD group, 3809 (88%) individuals had documented blood pressure monitoring. In comparison, 2462 (67%) individuals in the proxy-diagnosed CKD group and 9955 (80%) individuals in the undiagnosed CKD group had documented blood pressure monitoring. Annual monitoring of glucose, eGFR, and urine analysis results was conducted, and detailed data are presented in Table 2. Screening with UACR differed among groups, being documented in 50% of the diagnosed CKD group versus 20% in the proxy-diagnosed CKD group and 21% in the undiagnosed CKD group. eGFR monitoring was highest in the diagnosed CKD group (73%) and lowest in the proxy-diagnosed CKD group (53%).

**Fig. 1:**
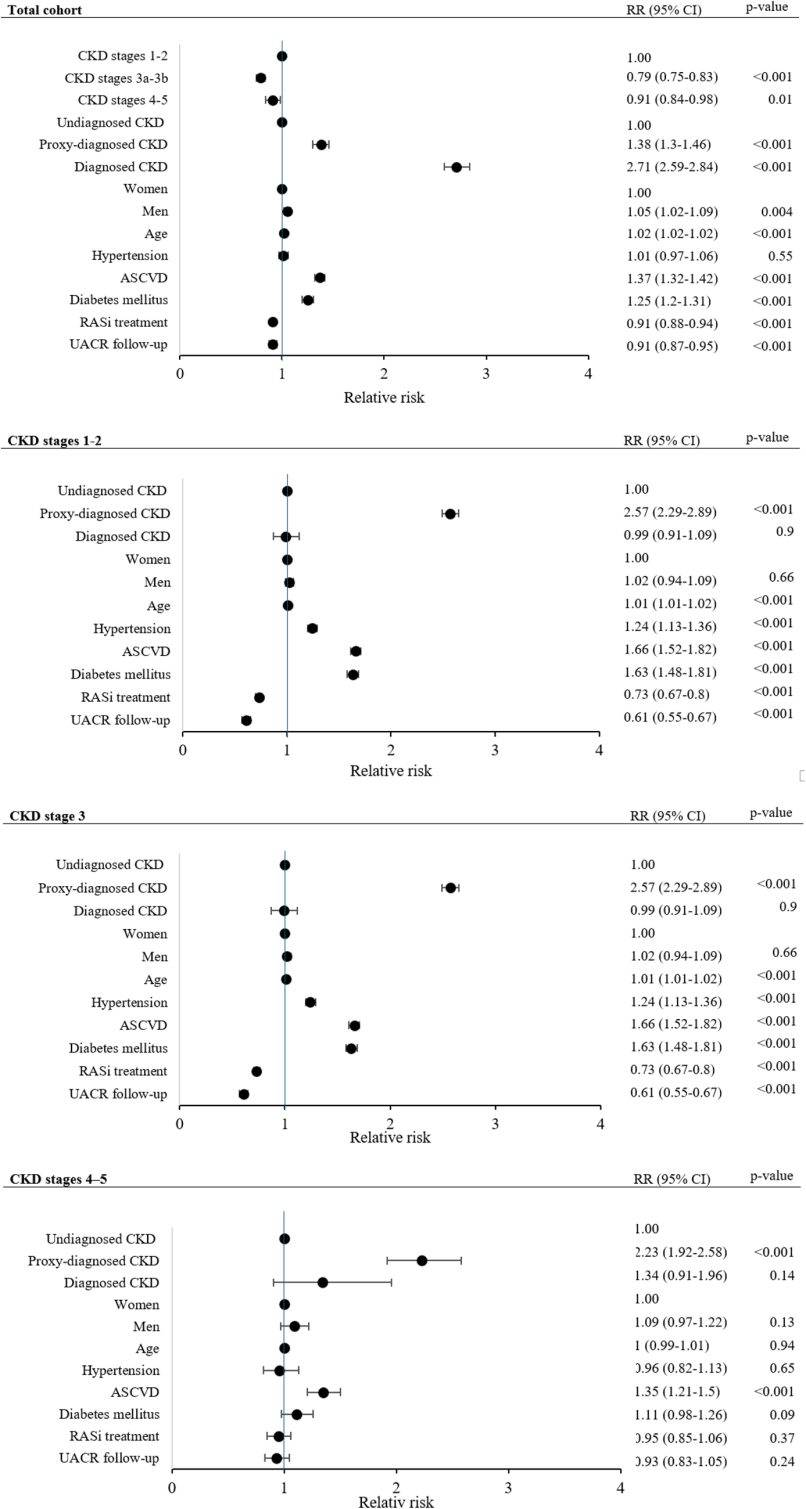
Forest plots of relative risk estimates from negative binomial regression models assessing hospitalization days in the total cohort and stratified by chronic kidney disease stages. *CKD* chronic kidney disease, *ASCVD* atherosclerotic cardiovascular disease, *RASi* renin-angiotensin system inhibitor, *UACR* urine albumin-to-creatinine ratio, *RR* relative risk, *CI* confidence interval

A stratified negative binomial regression analysis was conducted to evaluate hospital care days across CKD stages 1–2, 3, and 4–5. The results revealed that patients with diagnosed CKD or proxy-diagnosed CKD had a higher relative risk of hospitalization compared to those with undiagnosed CKD within each stage, as shown in Fig. 1. The analyses were adjusted for age, sex, CKD stage, and comorbidities including hypertension, ASCVD, and diabetes mellitus. In the total cohort, the adjusted relative risk of hospital care days for the diagnosed CKD group was 2.71 (95% CI: 2.59–2.84). Therapy with RASi was associated with a lower risk, with a relative risk of 0.91 (95% CI: 0.87–0.95). The groups with diagnosed CKD, proxy-diagnosed CKD, and undiagnosed CKD were analyzed separately using a negative binomial model for hospital days. The analysis, detailed in Supplementary Table 4, was adjusted for sex, age, comorbidities, RASi treatment, and UACR follow-up.

**Fig. 2:**
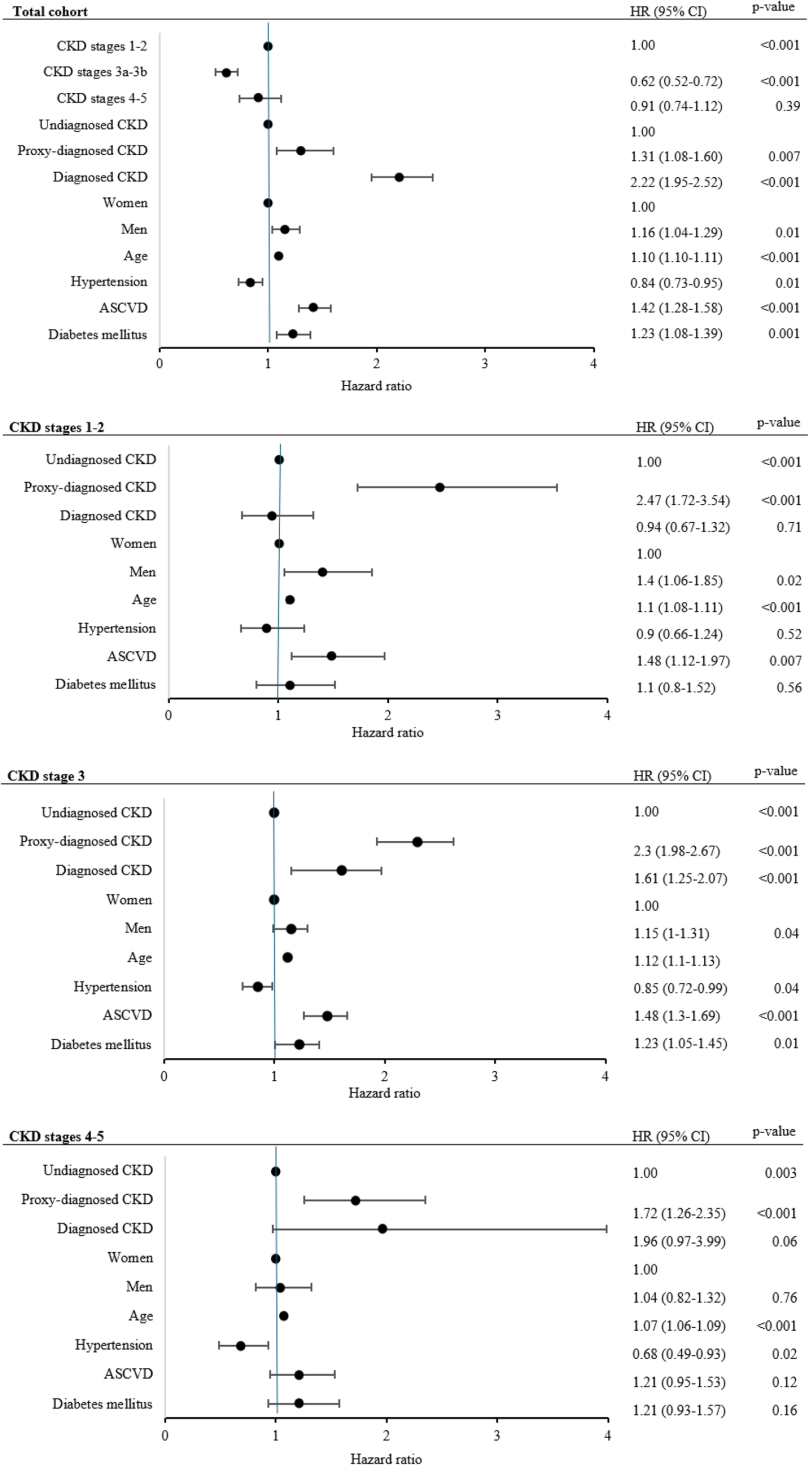
Forest plots of hazard ratio estimates from Cox proportional hazards regression models assessing all-cause mortality in the total cohort and stratified by chronic kidney disease stages. *CKD* chronic kidney disease, *ASCVD* atherosclerotic cardiovascular disease, *HR* hazard ratio, *CI* confidence interval

Cox regression of all-cause mortality was conducted, stratified by CKD stage. Compared to undiagnosed CKD, patients with diagnosed CKD had a hazard ratio (HR) of 2.22 (CI 1.95–2.52), while those with a proxy-diagnosed CKD had a corresponding HR of 1.31 (CI 1.08–1.60), as shown in Fig. 2. Stratified models were also estimated for CKD stages 1–2, 3, and 4–5, revealing that the strength and significance of the associations varied across CKD stages. A Cox regression model was used to analyze the HR for mortality; this was done separately for diagnosed CKD, proxy-diagnosed CKD, and undiagnosed CKD. The analysis was adjusted for sex, age, comorbidities, RASi treatment, and UACR follow-up, and is presented in Supplementary Table 4.

## Discussion

The present study reveals disparities in documentation among CKD patients. Within the cohort, 21% had diagnosed CKD, 18% proxy-diagnosed CKD, and 61% undiagnosed CKD. Patients with undiagnosed CKD were the oldest, followed by those with diagnosed CKD, while the proxy-diagnosed CKD group was the youngest. Patients with more advanced CKD stages and a higher burden of comorbidities were more likely to have diagnosed CKD and receive more comprehensive follow-up care. Both diagnosed CKD and proxy-diagnosed CKD were associated with increased hospitalization rates and greater healthcare utilization compared to undiagnosed CKD. Additionally, diagnosed CKD was associated with a higher risk of mortality than proxy-diagnosed CKD, even after adjusting for age and comorbidities.

A key contribution of this study is the identification of the proxy-diagnosed CKD group, which accounted for 18 percent of the cohort, nearly matching the proportion of formally diagnosed cases. This group likely reflects clinical recognition of kidney dysfunction without formal coding or management. Identifying this intermediate category highlights a gap in care and provides a more nuanced understanding of how CKD is documented and addressed in everyday clinical practice. CKD was diagnosed in 21% of the total cohort, which was slightly higher than the 18% or less reported in previous studies [3, 14]. The group with diagnosed CKD exhibited a higher prevalence of comorbidities and a greater number of individuals with CKD stage 3 to stage 5, which may contribute to improved diagnosis and management. Accurate recording of CKD starting from stage 3 is a crucial initial step in reducing disease progression and minimizing adverse outcomes [16, 23]. However, it is notable that 87% of individuals with undiagnosed CKD had CKD stage 3 or higher. Among those with CKD stages 3 to 5, only two-thirds underwent annual eGFR monitoring, compared to three-quarters in the diagnosed CKD group and approximately half of the patients in the proxy-diagnosed CKD group. This is still a relatively low percentage given the recommendations for annual check-ups [11, 13]. Only two-thirds of patients in the diagnosed CKD group underwent eGFR testing, and UACR testing was performed in just 50% of this group, with even lower rates in the other groups. Although UACR testing was higher in our cohort compared to other studies, these findings highlight the need for a more consistent approach to follow-up for this patient population [3, 24, 25].

RASi have been a cornerstone in the recommended treatment of CKD for many years [14]. Despite these guidelines, only 45% of the overall cohort were found to be using RASi, consistent with prior research. Among patients with diagnosed CKD, more than half were receiving RASi therapy, whereas usage was lower among those with undiagnosed CKD, and notably low (28%) in the proxy-diagnosed CKD group. Despite the proven benefits of RASi in reducing kidney failure, major cardiovascular events, and mortality in patients with CKD, its use has previously been reported at only 20.6% [26, 27]. A similar pattern was observed with statin use, which was highest among patients with a diagnosed CKD. This could be attributed to the higher incidence of ASCVD in this population and to guidelines recommending statin use for patients over 50 with CKD stages 3a to 5, regardless of cholesterol levels, if they are not on dialysis or post-kidney transplant [10, 28]. Since statin therapy was not recommended at the time of the study, there was no sub-analysis of patients eligible for statins. However, the use of RASi and statins has been associated with reduced all-cause mortality and hospital admissions in a previous study [14]. As of 2021, SGLT-2i have been recommended for CKD, but since the present study includes data referring to a period before these recommendations were implemented, it was not possible to draw any clear associations. SGLT-2i were most certainly prescribed primarily for managing diabetes, rather than for addressing renal impairment. Patients with a proxy-diagnosed CKD had lower rates of all the pharmacotherapies compared to other groups, underscoring the importance of receiving both an accurate and timely CKD diagnosis.

Individuals with a diagnosed CKD experienced more frequent hospital admissions and longer hospital stays compared to those with undiagnosed CKD or those having a proxy-diagnosed CKD. Healthcare utilization data revealed that hospital admissions and days of care were highest in the diagnosed CKD group, with rates more than double those of patients with undiagnosed CKD or with a proxy-diagnosed CKD. The relative risk for hospital days regarding the diagnosed CKD group was 2.71 (CI: 2.59–2.84), adjusted for age, sex, CKD stage, and comorbidities. This may suggest that patients were diagnosed at a more advanced stage of CKD, requiring more healthcare, or it may reflect improved recognition of CKD-related complications, prompting increased healthcare interventions. Nevertheless, these findings may partly reflect residual confounding, as diagnosed patients could represent a frailer subgroup with unmeasured factors, such as malnutrition or functional decline, influencing both healthcare use and outcomes. To further explore whether disease severity influenced the observed associations, stratified analyses by CKD stage were conducted for both hospitalization and mortality outcomes. These analyses confirmed that the associations between diagnosis status and outcomes persisted across CKD stages, although the strength of association varied. This supports the interpretation that diagnosis status itself, beyond CKD severity, is associated with differences in healthcare utilization and mortality risk. The proxy-diagnosed CKD group, consisting of younger patients with potentially earlier-stage CKD, had lower hospital admission rates and shorter hospital stays. The frequency of primary care visits showed minimal variation across all CKD groups. Even though a significant difference was observed, the number of primary care visits does not appear to be influenced to a greater extent by whether individuals have undiagnosed CKD, diagnosed CKD, or proxy-diagnosed CKD.

Previous studies have reported that mortality is associated with older age, proteinuria, and a higher prevalence of comorbidities such as ASCVD [29, 30]. The Cox regression model for all-cause mortality showed an HR of 2.22 (CI: 1.95-2.52) for patients with a formal diagnosed CKD and an HR of 1.31 (CI: 1.08-1.60) for those with a proxy-diagnosed CKD, even after adjusting for sex, age, CKD stages, and comorbidities. This suggests that a diagnosed CKD was associated with a greater number of comorbidities, such as ASCVD (HR: 1.42, CI: 1.28-1.39), and that the increased mortality risk is not necessarily driven solely by CKD, as previously reported [13]. Additionally, CKD, at stages 1 and 2, has been associated with an increased risk of both mortality and number of hospitalizations. This association was likely influenced by selection bias, as patients in these early stages often have comorbid conditions that impact these outcomes. Although this finding appears paradoxical, it has been observed in previous studies and cannot be explained by other factors [14]. In the proxy-diagnosed CKD group, the average age is notably lower, with a greater proportion of individuals in CKD stages 1-2 (60%) and a lower incidence of comorbidities. This may influence the findings regarding their need for medical care and potentially reduce the necessity for follow-up.

This study utilizes a comprehensive and well-established data source, RHIP, which provides a broad and detailed view of the CKD population within Region Halland. Moreover, the study design allows for the analysis of real-world data across various healthcare settings, including primary care, emergency departments, and specialized outpatient clinics, offering a holistic understanding of CKD management in routine clinical practice. The proxy-diagnosed CKD classification enables identification of at-risk individuals but may introduce heterogeneity due to the variable specificity of the included ICD-10 codes.

The retrospective nature of the research introduces inherent biases, such as the potential for incomplete or inaccurate ICD coding, which could underestimate the true prevalence of CKD. UACR values were not collected, which could in this context have been of value for assessing the CKD more extensively. The reliance on registry data limits the ability to capture all relevant clinical details, such as patient adherence to treatment or lifestyle factors, which could impact disease progression and outcomes. Despite adjustments for key variables such as age, sex, CKD stage, and major comorbidities, residual confounding may still be present. Unmeasured factors such as frailty, nutritional status, functional decline, or undiagnosed comorbid conditions could influence both the likelihood of receiving a CKD diagnosis and the risk of adverse outcomes. These limitations should be considered when interpreting associations between diagnosis status, healthcare utilization, and mortality. Furthermore, the study is geographically confined to Region Halland, which may limit the generalizability of the findings to other regions or countries with different healthcare systems and population characteristics. Lastly, while the study adjusts for several key variables, unmeasured confounding factors such as socioeconomic status or access to healthcare services may still influence the observed associations between CKD diagnosis and outcomes.

Additionally, since the study period covers only the calendar year 2019, the analysis reflects a time when clinical use of SGLT2 inhibitors was limited, which may affect the interpretation of treatment patterns and outcomes related to this therapy.

## Conclusion

This study reveals disparities in CKD diagnosis, with only 21% of patients having a diagnosed CKD. Compared to the proxy-diagnosed and undiagnosed CKD cohorts, the diagnosed CKD group was associated with more advanced stages of CKD, higher comorbidity burden, increased healthcare utilization and mortality, but better adherence to treatments like RASi and statins as well as more comprehensive follow-up and monitoring. In contrast, proxy-diagnosed CKD patients were younger, had less severe disease, and lower healthcare utilization. These patients also received less follow-up and monitoring and were undertreated which would mitigate further deterioration in kidney function. Prescription rates of recommended therapies were generally low, and gaps in monitoring and follow-up were particularly evident among proxy-diagnosed and undiagnosed CKD patients. These findings underscore the need for a more systematic approach to CKD diagnosis, follow-up, and management to improve outcomes and reduce disparities.

## Supplementary Information

Below is the link to the electronic supplementary material.

Supplementary file1 (PDF 78 KB)

Supplementary file2 (DOCX 41 KB)

## Data Availability

Data for this study were sourced from a third party and are not open to the public. The datasets created and examined in this study are kept confidential in line with the provisions of the Swedish Health and Medical Services Act concerning the Secrecy Act. Access to these datasets might be granted by RH after a formal request to the lead author and subsequent approval from the Regional Consultative Committee for Research Data in RH.
