# Supplementary material for: Differences in chronic kidney disease management based on identification and diagnosis in a population-based observational study
Source: J Nephrol. 2025 Sep 26;38(9):2809–20. doi: 10.1007/s40620-025-02414-2 (PMC12711941; doi:10.1007/s40620-025-02414-2)
Supplement: Supplementary file 1 — Supplementary file1 (PDF 78 KB) [file 40620_2025_2414_MOESM1_ESM.pdf]

Supplementary Figure 1. Flowchart of cohort selection for the chronic kidney disease study population.

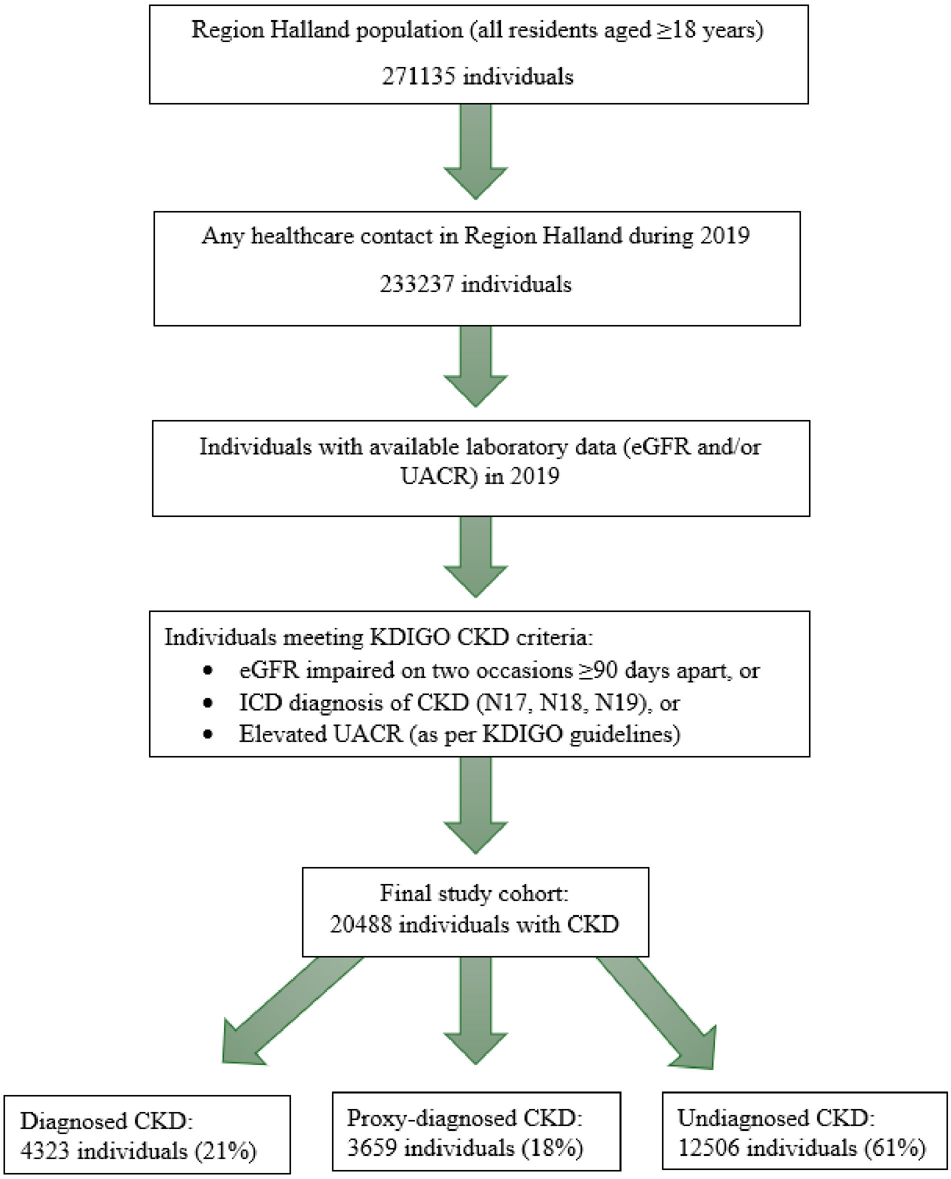

Footnotes: CKD: chronic kidney disease; eGFR: estimated glomerular filtration rate; UACR: urine albumin-to-creatinine ratio; ICD: International Classification of Diseases; KDIGO: Kidney Disease: Improving Global Outcomes.
