# Supplementary material for: Differences in chronic kidney disease management based on identification and diagnosis in a population-based observational study
Source: J Nephrol. 2025 Sep 26;38(9):2809–20. doi: 10.1007/s40620-025-02414-2 (PMC12711941; doi:10.1007/s40620-025-02414-2)
Supplement: Supplementary file 2 — Supplementary file2 (DOCX 41 KB) [file 40620_2025_2414_MOESM2_ESM.docx]

**Supplementary information**

Supplementary Table 1. KDIGO Classification of CKD by eGFR and Albuminuria Categories

| **KDIGO Stage** | **eGFR (mL/min/1.73m²)** | **CKD Description** | **Albuminuria**  **Category** | **UACR (mg/mmol)** |
| --- | --- | --- | --- | --- |
| **1** | ≥90 | Presupposes presence of other signs of kidney damage. eg albuminuria > 3 months. biopsy-verified kidney disease or polycystic kidney disease. | A1-A3 | <3–>30 |
| **2** | 60–89 | Presupposes presence of other signs of kidney damage. eg albuminuria > 3 months. biopsy-verified kidney disease or polycystic kidney disease. | A1-A3 | <3–>30 |
| **3a** | 45–59 | Mild to moderate renal impairment | A1-A3 | <3–>30 |
| **3b** | 30–44 | Moderate to severe renal impairment | A1-A3 | <3–>30 |
| **4** | 15–29 | Severe impaired renal function | A1-A3 | <3–>30 |
| **5** | <15 | Kidney failure | A1-A3 | <3–>30 |

Notes: KDIGO = Kidney Disease: Improving Global Outcomes; CKD = Chronic Kidney Disease; eGFR = Estimated Glomerular Filtration Rate (mL/min/1.73m²); UACR = Urine Albumin-to-Creatinine Ratio (mg/g or mg/mmol); eGFR = estimated glomerular filtration rate; A1 = Normal to Mildly Increased Albuminuria (UACR <30 mg/g or <3 mg/mmol); A2 = Moderately Increased Albuminuria (UACR 30–300 mg/g or 3–30 mg/mmol); A3 = Severely Increased Albuminuria (UACR >300 mg/g or >30 mg/mmol).

Supplementary Table 2. The criterias for chronic kidney disease based on ICD-10 diagnoses and coding rules.

| **Definitions** | **ICD-10 Codes** | **Diagnostic Terms** |
| --- | --- | --- |
| Diagnosed CKD | N17  N18  N19 | Acute kidney failure  Chronic kidney disease  Unspecified kidney failure |
| Proxy-diagnosed CKD | E102, E112, E122, E132, E142  I12, I13, I15.0, I15.1  N00–N08  N10–N16  Z992  Z49 | Diabetes with kidney complications  Hypertensive kidney disease  Glomerular disorders  Tubulo-interstitial nephritis  Dependence on renal dialysis  Care involving dialysis |
| Undiagnosed CKD | No CKD-related ICD diagnosis | KDIGO laboratory criteria (eGFR <60 mL/min/1.73m² on ≥2 occasions ≥90 days apart and/or elevated UACR). |

Classification Rules:

- Diagnosed CKD: Presence of any diagnosis code N17, N18, or N19 at least once between 2013 and 2019.
- Proxy-diagnosed CKD: Presence of any listed proxy ICD-10 codes above, without N17–N19
- Diagnosed CKD overrides proxy-diagnosed CKD if both are present.

Supplementary Table 3. Presenting the pharmacotherapies with ATC codes registered in the study.

| **Pharmacotherapy** | **Specific pharmacotherapy and ATC-code** | | | | | |
| --- | --- | --- | --- | --- | --- | --- |
| **ACEi** | Captopril  *C09AA01* | Enalapril  *C09AA02* | Lisinopril *C09AA03* | Perindopril *C09AA04* | Ramipril *C09AA05* |  |
| **ACEi + Thiazide** | Enalapril + Hydrochlorothiazide  *C09BA02* | Lisinopril + Hydrochlorothiazide *C09BA03* | Ramipril + Hydrochlorothiazide *C09BA05* |  |  |  |
| **ARB** | Losartan *C09CA01* | Valsartan  *C09CA03* | Irbesartan *C09CA04* | Candesartan *C09CA07* | Telmisartan *C09CA07* |  |
| **ARB +**  **Thiazide** | Losartan + Hydrochlorothiazide *C09DA01* | Valsartan + Hydrochlorothiazide *C09DA03* | Irbesartan + Hydrochlorothiazide *C09DA04* | Candesartan + Hydrochlorothiazide *C09DA06* | Telmisartan *C09DA07* |  |
| **ARB + calcium-antagonist** | Valsartan + Amlodipine *C09DB01* |  |  |  |  |  |
| **calcium-**  **antagonist** | Amlodipine  *C08CA01* | Felodipine  *C08CA02* | Nifedipine *C08CA05* | Lercanidipine *C08CA13* | Verapamil *C08DA01* | Diltiazem *C08DB01* |
| **Thiazide** | Bendroflumethiazide *C03AA01* | Hydrochlorothiazide *C03AA03* | Bendroflumethiazide + Kalium *C03AB01* |  |  |  |
| **Beta-**  **blockers** | Propranolol  *C07AA05* | Metoprolol *C07AB02* | Atenolol *C07AB03* | Bisoprolol *C07AB07* | Labetalol *C07AG01* | Carvedilol *C07AG02* |
| **Betablocker + Calcium-**  **antagonist** | Metoprolol + Felodipine *C07FB02* |  |  |  |  |  |
| **Statins** | Simvastatin  *C10AA01* | Rosuvastatin *C10AA03* | Atorvastatin *C10AA05* | Rosuvastatin *C10AA0* |  |  |
| **SGLT2i** | Empagliflozin  *A10BK03* | Dapagliflozin *A10BK01* |  |  |  |  |
| **Aldosterone-**  **antagonist** | Spironolactone  *C03DA01* |  |  |  |  |  |

Note: ACEi= angiotensin-converting enzyme inhibitor. ARB= angiotensin receptor blocker. SGLT2i=sodium-glucose cotransporter-2 inhibitor

Supplementary Table 4. Negative binomial model analyzing hospital days as the dependent variable, stratified by diagnosed CKD, proxy-diagnosed CKD, and undiagnosed CKD, adjusted for sex, age, comorbidities, RASi treatment, and UACR follow-up.

|  | **Diagnosed CKD** | | | | **Proxy-diagnosed CKD** | | | | **Undiagnosed CKD** | | | |
| --- | --- | --- | --- | --- | --- | --- | --- | --- | --- | --- | --- | --- |
|  | **RR** | **95% CI** | | **p-value** | **RR** | **95% CI** | | **p-value** | **RR** | **95% CI** | | **p-value** |
|  |  | **Lower** | **Upper** |  |  | **Lower** | **Upper** |  |  | **Lower** | **Upper** |  |
| *Basic of characteristics* | |  |  |  |  |  |  |  |  |  |  |  |
| Age | 1.06 | 1.05 | 1.08 | <0.001 | 1.02 | 1.01 | 1.02 | <0.001 | 1.03 | 1.02 | 1.03 | <0.001 |
| Women | 1.00 |  |  |  | 1.00 |  |  |  | 1.00 |  |  |  |
| Men | 1.01 | 0.85 | 1.2 | 0.94 | 1.07 | 0.98 | 1.17 | 0.16 | 1.1 | 1.05 | 1.15 | <0.001 |
| *CKD stages* |  |  |  |  |  |  |  |  |  |  |  |  |
| Stages 1-2 | 1.00 |  |  |  | 1.00 |  |  |  | 1.00 |  |  |  |
| Stages 3a-3b | 0.7 | 0.5 | 0.97 | <0.001 | 1.18 | 1.06 | 1.32 | 0.002 | 0.66 | 0.62 | 0.7 | <0.001 |
| Stages 4-5 | 1.12 | 0.8 | 1.58 | 0.5 | 1.24 | 0.86 | 1.78 | 0.24 | 0.81 | 0.71 | 0.94 | 0.004 |
| *Comorbidities* |  |  |  |  |  |  |  |  |  |  |  |  |
| Hypertension | 0.76 | 0.6 | 0.97 | 0.03 | 1.01 | 0.91 | 1.13 | 0.81 | 1.08 | 1.02 | 1.14 | 0.006 |
| ASCVD | 1.37 | 1.15 | 1.63 | <0.001 | 1.41 | 1.261 | 1.576 | <0.001 | 1.32 | 1.26 | 1.39 | <0.001 |
| Diabetes mellitus | 1.79 | 1.48 | 2.17 | <0.001 | 1.3 | 1.15 | 1.47 | <0.001 | 1.16 | 1.08 | 1.24 | <0.001 |
| *Follow-up* |  |  |  |  |  |  |  |  |  |  |  |  |
| RASi treatment | 0.86 | 0.72 | 1.02 | 0.08 | 0.87 | 0.78 | 0.97 | 0.01 | 0.88 | 0.84 | 0.92 | <0.001 |
| UACR | 0.37 | 0.29 | 0.46 | <0.001 | 0.83 | 0.73 | 0.94 | 0.003 | 0.94 | 0.88 | 1.01 | 0.08 |

Note: RR=relative risk, CI=confidence interval , CKD=chronic kidney disease, ASCVD=atherosclerotic cardiovascular disease, RASi= renin-angiotensin system inhibitors, UACR=urine albumin-creatinine ratio.

Supplementary Table 5. Cox regression analysis of mortality, stratified by diagnosed CKD, proxy-diagnosed CKD, and undiagnosed CKD, adjusted for sex, age, comorbidities, RASi treatment, and UACR follow-up.

|  | **Diagnosed CKD** | | | | **Proxy-diagnosed CKD** | | | | **Undiagnosed CKD** | | | |
| --- | --- | --- | --- | --- | --- | --- | --- | --- | --- | --- | --- | --- |
|  | **HR** | **95% CI** | |  | **HR** | **95% CI** | |  | **HR** | **95% CI** | |  |
|  |  | **Lower** | **Upper** | **p-value** |  | **Lower** | **Upper** | **p-value** |  | **Lower** | **Upper** | **p-value** |
| *Basic of characteristics* | |  |  |  |  |  |  |  |  |  |  |  |
| Age | 1.06 | 1.05 | 1.08 | <0.001 | 1.08 | 1.06 | 1.1 | <0.001 | 1.12 | 1.11 | 1.13 | <0.001 |
| Women | 1.00 |  |  |  | 1.00 |  |  |  | 1.00 |  |  | <0.001 |
| Men | 1.01 | 0.85 | 1.2 | 0.94 | 1.06 | 0.75 | 1.51 | 0.73 | 1.35 | 1.17 | 1.57 |  |
| *CKD stages* |  |  |  |  |  |  |  |  |  |  |  |  |
| CKD stages 1-2 | 1.00 |  |  | <0.001 | 1.00 |  |  | 0.05 | 1.00 |  |  | <0.001 |
| CKD stages 3a-3b | 0.7 | 0.5 | 0.97 | 0.03 | 1.12 | 0.74 | 1.67 | 0.6 | 0.54 | 0.44 | 0.67 | <0.001 |
| CKD stages 4-5 | 1.12 | 0.8 | 1.58 | 0.5 | 2.5 | 1.18 | 5.34 | 0.02 | 0.86 | 0.61 | 1.21 | 0.38 |
| *Comorbidities* |  |  |  |  |  |  |  |  |  |  |  |  |
| Hypertension | 0.76 | 0.6 | 0.97 | 0.03 | 1.17 | 0.77 | 1.78 | 0.46 | 0.92 | 0.77 | 1.1 | 0.36 |
| ASCVD | 1.37 | 1.15 | 1.63 | <0.001 | 1.27 | 0.78 | 2.07 | 0.33 | 1.42 | 1.22 | 1.65 | <0.001 |
| Diabetes mellitus | 1.79 | 1.48 | 2.17 | <0.001 | 1.42 | 0.99 | 2.05 | 0.06 | 1.82 | 1.49 | 2.23 | <0.001 |
| *Follow-up* |  |  |  |  |  |  |  |  |  |  |  |  |
| RASi treatment | 0.86 | 0.72 | 1.02 | 0.08 | 0.85 | 0.58 | 1.26 | 0.42 | 0.99 | 0.85 | 1.15 | 0.90 |
| UACR | 0.37 | 0.29 | 0.46 | <0.001 | 0.18 | 0.08 | 0.43 | <0.001 | 0.29 | 0.21 | 0.4 | <0.001 |

Note: HR=hazard ratio, CI=confidence interval, CKD=chronic kidney disease, ASCVD=atherosclerotic cardiovascular disease, RASi= renin-angiotensin system inhibitors, UACR=urine albumin-creatinine ratio.
